# An arterial map of the uterine artery - a tool for endovascular and gynecological procedures

**DOI:** 10.1007/s00276-024-03387-w

**Published:** 2024-05-31

**Authors:** Monika Konarska-Włosińska, Alicia Del Carmen Yika, Martyna Dziedzic, Michał Bonczar, Patryk Ostrowski, Wadim Wojciechowski, Jerzy Walocha, Mateusz Koziej

**Affiliations:** 1 Department of Anatomy, Jagiellonian University Medical College Cracow, Mikołaja Kopernika 12, Kraków, 33-332 Poland; 2 Youthoria, Youth Research Organization, Kraków, Poland; 3 Department of Radiology, Jagiellonian University Medical College Cracow, Kraków, Poland

**Keywords:** Uterine artery, Uterus, Embolization, Anatomy, Surgery

## Abstract

**Introduction:**

The anatomy of the uterine artery (UA) is highly complex, demonstrating various patterns of origin and course. The main objective of the present study is to provide the first anatomical heat map of the UA, demonstrating the location of its origin and course in the pelvis.

**Methods:**

In July 2022, an assessment was conducted on the findings from 40 consecutive female patients who had undergone computed tomography angiography of the abdomen and pelvis. Morphometric features of the UA and its associated anatomical area were gathered in 19 categories.

**Results:**

The presented results are based on a total of 58 UAs. 40 UAs originated from the anterior trunk of the internal iliac artery (69.0%), 16 of the UAs originated from the umbilical artery (27.6%), and the remaining two originated from the inferior gluteal artery (3.4%). The median diameter of the UA at its origin was found to be 3.20 mm (LQ = 2.63; HQ = 3.89).

**Conclusion:**

The anatomy of the UA is highly complex, showcasing variable topography, origin patterns, and morphometric properties. In the present study, a novel arterial map of this vessel was made, highlighting the diversity in its origin location and course. In our studied cohort, the UA originated most commonly from the anterior trunk of the internal iliac artery (69.0%), as described in the major anatomical textbooks. Having adequate knowledge about the anatomy of this artery is of immense importance in various gynecological and endovascular procedures, such as hysterectomies and embolizations.

## Introduction

The anatomy of the uterine artery (UA) is highly complex, demonstrating various patterns of origin and course. It originates from the anterior trunk of the internal iliac artery and is the counterpart of the artery associated with the ductus deferens in males. It follows a course along the lateral pelvic wall, positioning itself anterior to the internal iliac artery. The UA proceeds medially until it reaches the junction of the uterus and vagina. Once it reaches the side of the cervix, the UA divides into two branches: a smaller descending vaginal branch that supplies the cervix and vagina and a larger ascending branch that runs along the lateral border of the uterus, providing it with blood. Subsequently, the ascending branch splits into ovarian and tubal branches, which supply the inner parts of the ovary and uterine tube, respectively, and establish anastomoses with the ovarian and tubal branches of the ovarian artery. Moreover, during its course, the UA crosses over the ureter [12].

The arterial anatomy of the pelvis is quite variable [9, 19–21]. This variability was showcased in a recent meta-analysis [13], which examined the origin and morphometric properties of the UA. Although the major anatomical textbooks describe the UA’s origin as being from the anterior trunk of the internal iliac artery, many other origins have been presented in the literature. The vessel has been reported to also originate from the umbilical artery, the inferior gluteal artery, and the internal pudendal artery, amongst others [2, 4]. Moreover, numerous studies have analyzed the vessel’s clinically relevant morphometric properties, such as its length and diameter. This knowledge is especially important in endovascular procedures, such as during the embolization of the UA as a treatment for pelvic hemorrhage or uterine fibroids [14, 16, 18]. However, no study has analyzed the precise topography of the UA, providing surgeons with a useful tool for visualizing the course and origin of the UA pre- and intraoperatively in various pelvic surgeries. Therefore, the main objective of the present study is to provide the first anatomical heat map of the UA, demonstrating the location of its origin and course in the pelvis.

## Materials and methods

### Approvement of the bioethical committee

The research protocol was submitted for evaluation and approved by the Jagiellonian University Bioethical Committee, Cracow, Poland (1072.6120.254.2022). The research was conducted in accordance with the allowed criteria throughout the subsequent phases.

### Study group

In July 2022, an assessment was conducted on the findings from 40 consecutive female patients who had undergone computed tomography angiography (CTA) of the abdomen and pelvis at the Radiology Department of Jagiellonian University Medical College in Cracow, Poland. Each CTA was evaluated bilaterally; therefore, a total of 80 sides were initially evaluated. The exclusion criteria were defined as (1) trauma to the abdominal or pelvic region that could impact the structure or dimensions of the UA or its nearby anatomy, (2) substantial artifacts hindering the accurate imaging and measurement of the UA or its adjacent anatomical region, (3) poor-quality and unreadable images, and (4) significant lack of contrast filling the whole arterial system. If any of the mentioned defects only impacted half of the CTA without affecting the contralateral side, the other UA was assessed independently. The great majority (*n* = 19) of the excluded sides were not analyzed due to significant artifacts. The other three were disqualified to prevent bias, given their images’ poor quality. Finally, a total of 58 sides met the inclusion criteria. None of the analyzed CTAs contained significant pathological changes that could substantially displace any of the studied structures. Therefore, a total of 58 UAs were analyzed.

### Results acquisition

All CTAs of the abdomen and pelvis were performed on a 128-slice scanner CT (Philips Ingenuity CT, Philips Healthcare). The main CT imaging parameters were the following: collimation/increase: 0.625 / 0.3 mm; tube current: 120 mAs; field of view: 210 mm; matrix size: 512 × 512.

All patients received intravenous administration of contrast material at a dose of 1 ml/kg (standard dose). A non-ionic contrast medium (CM) containing 350 mg of iodine per ml was used (Jowersol 741 mg/ml, Optiray®, Guerbet, France). The acquisition of CT data was initiated using a real-time bolus tracking technique (Philips Healthcare), with the region of interest (ROI) placed in the descending aorta. CM was injected intravenously using a power injector at a flow rate of 5 ml / s. This was immediately followed by injecting 40 ml of saline solution at the same flow rate. Following injection of CM and saline, image acquisition was automatically started with a 2 s delay when the attenuation trigger value reached a threshold of 120 Hounsfield units (HU). Scanning was performed in the caudocranial direction.

The CTAs were analyzed on a dedicated workstation in the Anatomical Department of Jagiellonian University Medical College, Cracow, Poland. To ensure the highest possible quality of the visualizations and measurements and minimize potential bias, Materialise Mimics Medical version 21.0 software (Materialise NV, Leuven, Belgium) software was used. 3-dimensional (3D) reconstructions of each scan were developed, employing a set of settings, severally adjusted to each scan.

### Evaluation and measurements

At the beginning of every examination, each UA has been completely visualized. Following that, a series of measurements of each UA were taken by two separate researchers, and an average was calculated by considering both sets of results. All measurements were rounded to two decimal places. Morphometric features of the UA and its associated anatomical area were gathered in 19 categories: (1) UA diameter at its origin ; (2) UA cross-sectional area at its origin ; (3) UA angle at its origin [deg] ; (4) diameter of the anterior trunk of the internal iliac artery near the origin of the UA ; (5) cross-sectional area of the anterior trunk of the internal iliac artery near the origin of the UA ; (6) distance from the origin of the anterior trunk of the internal iliac artery to the origin of the UA ; (7) distance from the obturator artery to the UA ; (8) distance from the middle anorectal artery to the UA ; (9) distance from the superior vesical artery to the UA ; (10) distance from the vaginal artery to the UA ; (11) distance from the inferior gluteal artery to the UA ; (12) distance from the internal pudendal artery to the UA ; (13) number of branches that originate from the UA ; (14) distance from the origin of the UA to the origin of the ascending branch of the UA ; (15) diameter of the ascending branch at its origin ; (16) cross-sectional area of the ascending branch at its origin ; (17) distance from the origin of the UA to the origin of the descending branch of the UA ; (18) diameter of the descending branch at its origin ; (19) cross-sectional area of the descending branch at its origin. Additionally, patients’ uterus length [mm] has also been studied. The results were established in millimeters (mm / mm^2^). Nevertheless, after initial statistical evaluation, distances from the superior vesical artery and the vaginal artery to the UA were excluded from the analyses due to significant heterogeneity of the results and bias prevention.

Furthermore, a set of measurements was taken to establish an anatomical heat map of the occurrence of the origin of the UA. Using these measurements, a uniform anatomical triangle was defined. Subsequently, the shortest distances from the origins of each UA to the sides of the triangle were determined. All measurements were taken at a fixed angle to minimize potential bias. Furthermore, the points of origin of each UA were scaled and applied to the heatmap with respect to the enrolled measurements.

### Statistical analysis

Statistical analysis was performed with STATISTICA v13.1 (StatSoft Inc., Tulsa, OK, USA). The frequency and percentages presented qualitative features. The Shapiro-Wilk test was used to assess the normal distribution. Quantitative characteristics were presented by medians and upper and lower quartiles (UQ, LQ), as well as means and standard deviation (SD), depending on the verified normality of the data. Statistical significance was defined as *p* ≤ 0.05. Mann-Whitney and Wilcoxon signed-rank tests were used to establish potential differences between groups. The Spearman rank correlation coefficient was used to determine possible correlations between the parameters.

## Results

**Fig. 1:**
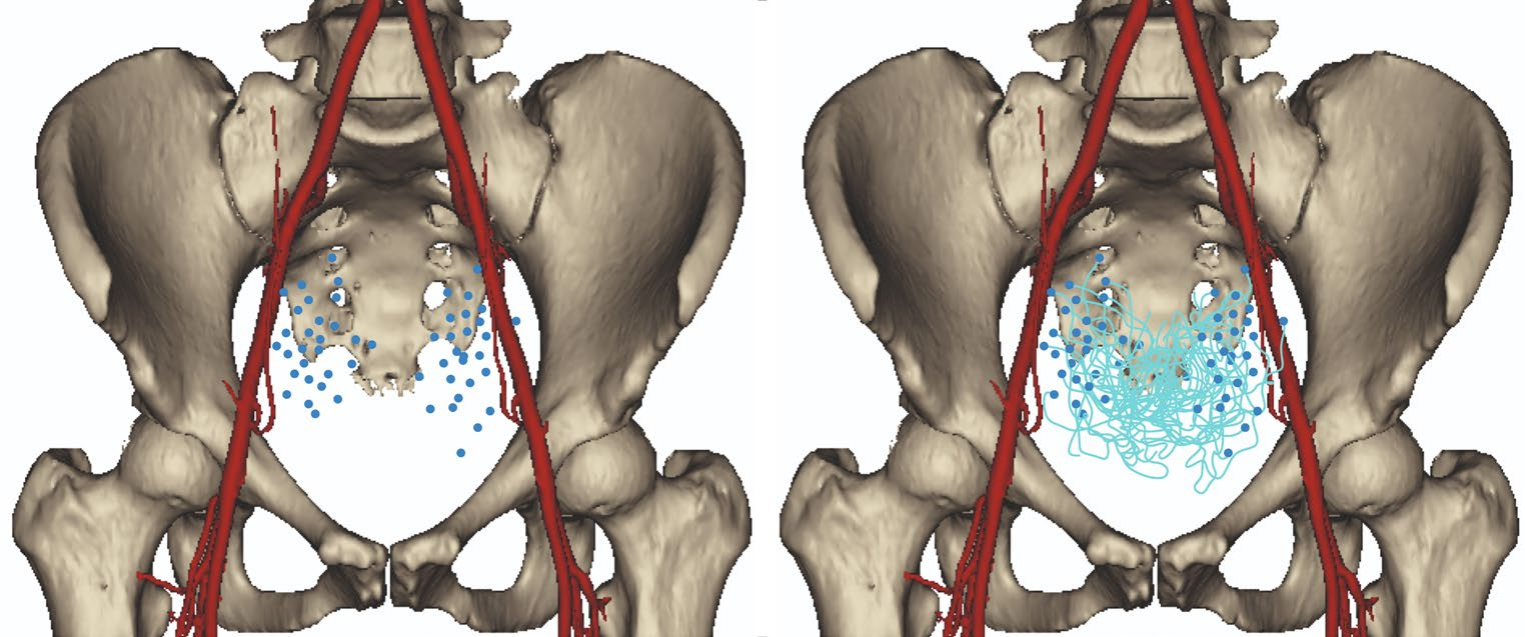
Heatmap presenting the occurance of the origin of the Uterine Artery and its course

**Fig. 2:**
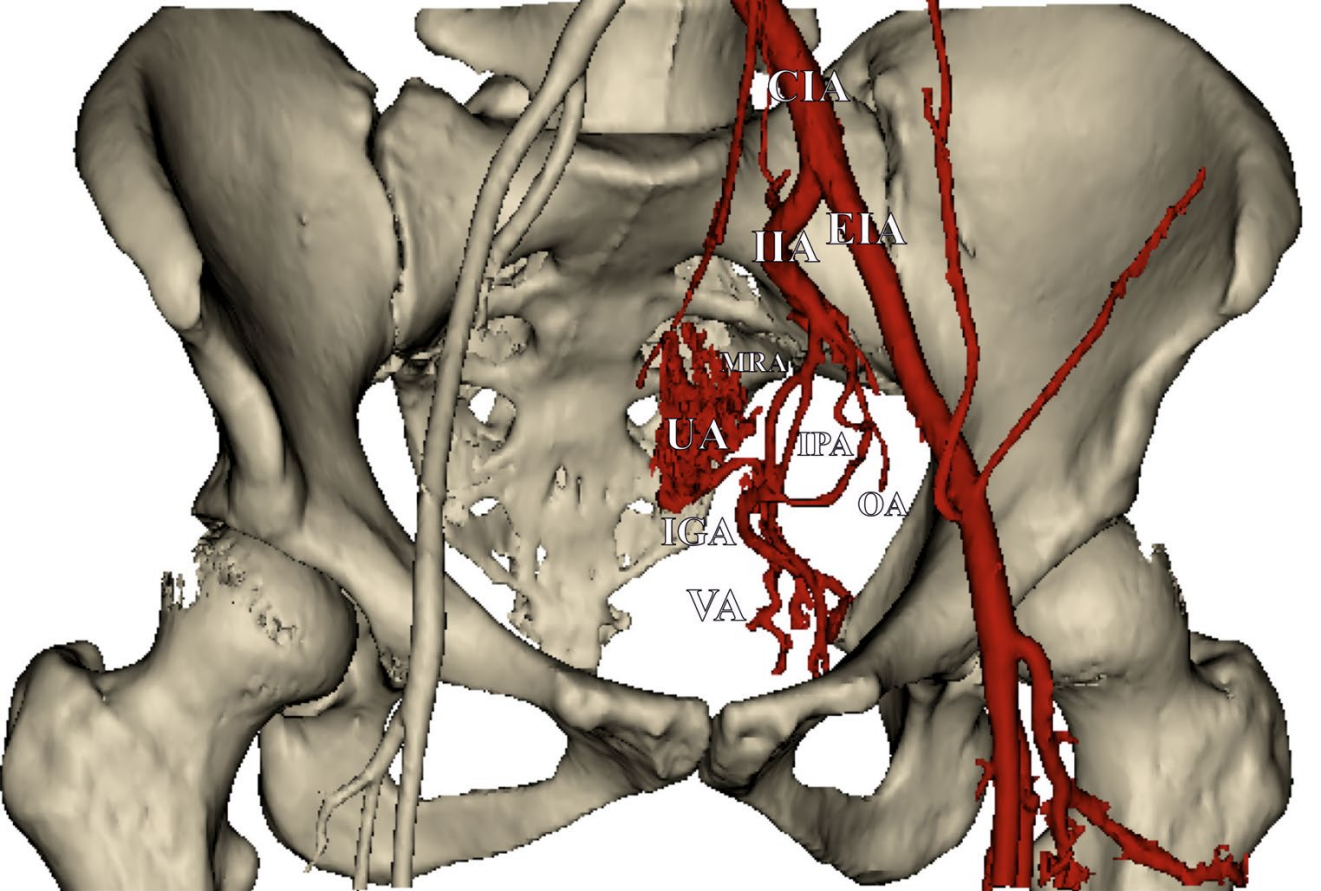
A three-dimensional, computed tomography angiography of the studied area. Some of the arteries were removed in order to provide beter visibility. UA - Uterine Artery. CIA - Common Iliac Artery. EIA - External Iliac Artery. IIA - Internal Iliac Artery. IGA - Inferior Gluteal Artery. IPA - Internal Pudendal Artery. OA - Obturator Artery. VA - Vaginal Artery. MRA - Middle Rectal Artery

The presented results are based on a total of 58 UAs. The mean age of the patients was 47.1 years old (SD = 15.4; Min = 24.0; Max = 77.0). All further data refer to the number of sides instead of number of patients. Left and right sides were analyzed in equal amounts, twenty-nine each. Forty UAs originated from the anterior trunk of the internal iliac artery (69.0%), 16 of the UAs originated from the umbilical artery (27.6%), and the remaining two originated from the inferior gluteal artery (3.4%). The anatomical heat map of the origin of the UA from the anterior point of view is presented in Fig. 1. The origin points and course of the studied UAs were scaled and appropriately applied into this figure. Figure 2 illustrates the arterial anatomy of the pelvis, highlighting the topographical characteristics of the UA.

The median diameter of the UA at its origin was found to be 3.20 mm (LQ = 2.63; HQ = 3.89). Moreover, the medial cross-sectional area at its origin was set to be 6.04 mm^2^ (LQ = 4.65; HQ = 6.48). UA’s median departure angle was set to be 130.47 degrees (LE = 116.44; HQ = 145.33). The distances between each branch of the anterior trunk of the internal iliac artery were measured over the surface of the said artery. The median distance from the anterior trunk of the internal iliac artery’s origin to the UA’s origin was set to be 17.21 mm (LQ = 13.63; HQ = 20.91). Furthermore, the median distance from the obturator artery to the UA was established at 13.35 mm (LQ = 8.76; HQ = 16.89). The median distance from the middle anorectal artery to the UA was set to be 26.13 (LQ = 18.55; HQ = 33.87). Moreover, the median distance from the inferior gluteal artery to the UA was found to be 45.96 mm (LQ = 34.67; HQ = 56.21). The median distance from the internal pudendal artery to the UA was set to be 12.14 mm (LQ = 8.22; HQ = 30.67). The mean number of branches of the UA was set to be 1.86 (SD = 0.35). Detailed results in each category are presented in Table 1.

**Table 1:** Results of the measurements. LQ – lower quartile. HQ – higher quartile. SD – standard deviation. UA – Uterine Artery. ATIIA – Anterior Trunk of the Internal Iliac Artery. OA – Obturator Artery. MRA – Middle Rectal Artery. IGA – Inferior Gluteal Artery. IPA – Internal Pudendal Artery

Category	Median	LQ	HQ	Minimum	Maximum	Mean	SD
UA Diameter at its Origin [mm]	3.20	2.63	3.89	2.00	5.69	3.37	0.91
UA Cross-sectional Area at its Origin [mm^2^]	6.04	4.65	6.48	3.64	7.92	5.71	1.14
UA Angle at its Origin [deg]	130.47	116.44	145.33	106.61	167.45	132.19	17.14
Diameter of the ATIIA near the origin of the UA [mm]	6.06	5.36	6.60	4.26	8.93	6.06	1.15
Cross-sectional Area of the ATIIA near the origin of the UA [mm^2^]	23.38	18.24	27.65	15.33	33.91	23.07	5.33
Distance from the origin of the ATIIA to the origin of the UA [mm]	16.17	13.75	21.54	10.20	32.73	17.41	5.14
Distance from the OA to the UA [mm]	13.35	8.76	16.89	4.12	21.65	12.73	4.97
Distance from the MRA to the UA [mm]	26.13	18.55	33.87	13.22	58.29	27.47	9.74
Distance from the IGA to the UA [mm]	45.96	34.67	56.21	17.01	72.71	46.44	13.78
Distance from the IPA to the UA [mm]	12.14	8.22	30.67	5.24	49.99	18.97	14.00
How many branches take off from the UA?	2.00	2.00	2.00	1.00	2.00	1.86	0.35
Distance from the origin of the UA to the origin of the Ascending Branch of the UA [mm]	68.12	55.55	74.83	41.78	95.60	65.59	12.08
Diameter of the Ascending Branch at its origin [mm]	3.67	3.41	4.21	3.01	4.87	3.77	0.51
Cross-sectional area of the Ascending Branch at its origin [mm^2^]	5.02	4.21	5.90	3.00	7.72	5.00	1.10
Distance from the origin of the UA to the origin of the Descending Branch of the UA [mm]	69.13	58.92	74.14	41.13	96.82	66.83	11.13
Diameter of the Descending Branch at its origin [mm]	3.19	2.58	3.64	2.00	4.78	3.15	0.66
Cross-sectional area of the Descending Branch at its origin [mm^2^]	3.64	3.30	3.89	3.00	5.11	3.71	0.53
Uterus Length [mm]	68.42	61.55	74.62	50.92	83.19	68.00	9.25

Furthermore, each parameter has been analyzed to find potential side-related statistical differences. Nevertheless, none of the parameters significantly differed in relation to the studied side. Detailed results regarding this part of the analysis are demonstrated in Table 2.

**Table 2:** Results of the measurements concerning the patients’ side. LQ – lower quartile. HQ – higher quartile. SD – standard deviation. UA – Uterine Artery. ATIIA – Anterior Trunk of the Internal Iliac Artery. OA – Obturator Artery. MRA – Middle Rectal Artery. IGA – Inferior Gluteal Artery. IPA – Internal Pudendal Artery. P-values were established using U Mann-Whitney test. P-values greater than 0.05 were considered as statistically significant

Category	Side	Median	LQ	HQ	Minimum	Maximum	Mean	SD	*P* value
UA Diameter at its Origin [mm]	Right	3.33	2.63	3.97	2.21	5.69	3.40	0.91	0.88
	Left	3.12	2.70	3.74	2.00	5.62	3.33	0.93	
UA Cross-sectional Area at its Origin [mm^2^]	Right	6.27	4.68	6.57	3.64	7.92	5.74	1.27	0.59
	Left	6.00	4.65	6.32	3.89	7.41	5.69	1.00	
UA Angle at its Origin [deg]	Right	136.66	114.66	145.34	106.61	167.45	132.39	18.53	0.91
	Left	130.27	118.75	143.62	109.72	165.71	131.98	15.95	
Diameter of the ATIIA near the origin of the UA [mm]	Right	6.28	5.02	6.52	4.54	8.93	6.10	1.27	0.92
	Left	5.94	5.40	6.75	4.26	7.98	6.02	1.03	
Cross-sectional Area of the ATIIA near the origin of the UA [mm^2^]	Right	23.15	17.48	25.56	15.33	32.39	22.41	5.30	0.35
	Left	24.10	18.48	27.88	15.69	33.91	23.73	5.41	
Distance from the origin of the ATIIA to the origin of the UA [mm]	Right	15.69	13.40	20.28	10.52	32.73	17.38	5.52	0.86
	Left	17.21	13.99	21.54	10.20	26.91	17.44	4.88	
Distance from the OA to the UA [mm]	Right	13.39	6.89	15.02	4.12	21.65	12.17	5.09	0.57
	Left	13.29	9.86	17.15	4.97	20.85	13.30	4.93	
Distance from the MRA to the UA [mm]	Right	23.67	17.98	36.56	14.34	50.32	26.89	10.11	0.60
	Left	26.46	18.90	33.87	13.22	58.29	28.06	9.52	
Distance from the IGA to the UA [mm]	Right	44.76	34.21	56.02	27.52	70.67	46.39	13.79	0.84
	Left	47.15	36.20	56.21	17.01	72.71	46.49	14.01	
Distance from the IPA to the UA [mm]	Right	11.74	7.55	32.64	5.24	48.55	19.32	15.23	0.70
	Left	12.33	9.22	26.98	6.55	49.99	18.62	12.92	
How many branches take off from the UA?	Right	2.00	2.00	2.00	1.00	2.00	1.86	0.35	0.99
	Left	2.00	2.00	2.00	1.00	2.00	1.86	0.35	
Distance from the origin of the UA to the origin of the Ascending Branch of the UA [mm]	Right	67.89	55.23	75.13	41.78	84.75	64.61	12.00	0.60
	Left	70.33	57.27	74.63	45.43	95.60	66.57	12.29	
Diameter of the Ascending Branch at its origin [mm]	Right	3.58	3.23	4.32	3.01	4.87	3.79	0.61	0.77
	Left	3.68	3.54	3.89	3.02	4.80	3.74	0.40	
Cross-sectional area of the Ascending Branch at its origin [mm^2^]	Right	5.03	4.24	6.00	3.03	7.72	5.03	1.19	0.80
	Left	5.01	4.21	5.80	3.00	7.03	4.97	1.04	
Distance from the origin of the UA to the origin of the Descenging Branch of the UA [mm]	Right	68.15	57.21	74.23	41.13	84.89	65.94	11.04	0.67
	Left	70.89	60.12	73.56	45.49	96.82	67.72	11.38	
Diameter of the Descending Branch at its origin [mm]	Right	3.16	2.58	3.67	2.00	4.78	3.13	0.71	0.88
	Left	3.20	2.66	3.57	2.19	4.37	3.16	0.62	
Cross-sectional area of the Descending Branch at its origin [mm^2^]	Right	3.58	3.30	3.89	3.01	5.11	3.69	0.52	0.92
	Left	3.65	3.43	3.86	3.00	5.00	3.73	0.54	

Subsequently, potential correlations between the analyzed parameters, the patient’s age, the UA origin diameter, and uterus length have been investigated. Statistically significant correlations were found between patients’ age and UA diameter at its origin, the UA cross-sectional area at its origin, the cross-sectional area of the anterior trunk of the internal iliac artery near the origin of the UA, the distance from the obturator artery to the UA, and the cross-sectional area of the ascending branch at its origin. Every aforementioned parameter decreased with age. Only the diameter of the ascending branch at its origin statistically significantly correlated with the uterus length (*R* = -0.30). The complete correlation analysis is presented in Table 3.

**Table 3:** Correlations between the measured parameters and patient’s age and the Diameter of the Uterine Artery (UA) at its origin. Highlighted in red are those in which the p-value was less than 0.05. R - Spearman’s Correlation Test was used in this statistical analysis. ATIIA – Anterior Trunk of the Internal Iliac Artery. OA – Obturator Artery. MRA – Middle Rectal Artery. IGA – Inferior Gluteal Artery. IPA – Internal Pudendal Artery

Category	Spearman’s *R*		
	Patient’s age	UA Origin Diameter	Uterus Length
UA Diameter at its Origin [mm]	-0.38	-	0,11
UA Cross-sectional Area at its Origin [mm^2^]	-0.27	0.61	0,00
UA Angle at its Origin [deg]	-0.20	-0.04	0,03
Diameter of the ATIIA near the origin of the UA [mm]	-0.26	-0.21	0,15
Cross-sectional Area of the ATIIA near the origin of the UA [mm^2^]	-0.48	0.19	0,16
Distance from the origin of the ATIIA to the origin of the UA [mm]	-0.06	0.03	0,30
Distance from the OA to the UA [mm]	-0.45	0.10	0,02
Distance from the MRA to the UA [mm]	0.14	-0.04	-0,22
Distance from the IGA to the UA [mm]	0.02	-0.07	-0,04
Distance from the IPA to the UA [mm]	-0.09	0.06	-0,02
How many branches take off from the UA?	0.11	0.12	-0,17
Distance from the origin of the UA to the origin of the Ascending Branch of the UA [mm]	-0.02	0.36	0,15
Diameter of the Ascending Branch at its origin [mm]	-0.07	0.11	-0,30
Cross-sectional area of the Ascending Branch at its origin [mm^2^]	-0.30	0.66	0,04
Distance from the origin of the UA to the origin of the Descending Branch of the UA [mm]	-0.12	0.31	0,19
Diameter of the Descending Branch at its origin [mm]	-0.06	-0.16	0,25
Cross-sectional area of the Descending Branch at its origin [mm^2^]	-0.12	0.13	0,11
Uterus Length [mm]	-0.54	0.11	-

## Discussion

The variable anatomy of the UA has been discussed extensively in the literature due to its relevance in various gynecological and endovascular procedures. Although this vessel is described as a branch of the anterior trunk of the internal iliac artery, many other origins have been presented [13]. Historically, the studies of Lipshutz [11], Adachi [1], Ashley and Anson [3], and Gasparri and Brizzi [5] provided us with the fundamental knowledge concerning the anatomy of the UA. More recently, in a study about the relevance of computed tomography angiographies in finding anatomical variations of the origin of the said vessel, Hao et al. [7] demonstrated that the artery originates most commonly from the inferior gluteal artery (64.3%). Interestingly, Arfi et al. [2] demonstrated that the umbilical artery is the dominant origin for the UA (62.7%). This was also demonstrated by Holub et al. [8]. Remarkably, the study did not report any cases of the UA originating directly from the internal iliac artery. Our study, which was based on 58 angiographies, shows that the UAs originated most commonly from the anterior trunk of the internal iliac artery (69.0%). However, the UA originated relatively frequently from the umbilical artery (27.6%) and rarely from the inferior gluteal artery (3.4%). Ultimately, our results support those presented in the literature, as they exhibit similarities with the recent meta-analysis conducted by Ostrowski et al. [13], where they presented the origin from the anterior trunk of the internal iliac artery as the most frequent one. However, controversy surrounds the studies that identified the inferior gluteal artery as the primary source of the UA [6, 7]. The inferior gluteal artery is typically characterized as the terminal branch of the internal iliac artery. Consequently, in instances where the UA emerges as the final branch of the anterior division of the internal iliac artery before termination, certain authors might classify it as originating from the inferior gluteal artery rather than from the anterior division of the internal iliac artery. This discrepancy may create bias regarding the definition of the origin of the UA. As mentioned earlier, the umbilical artery has also been demonstrated to be a frequent origin point of the UA [2]. Ultimately, this variability in the origin has been said to be linked with the population studied, showing a significantly high prevalence of variations in the Caucasian population [2, 4].

The internal iliac arteries originate from the fetal umbilical arteries during embryological development. Initially, the dorsal aorta undergoes bifurcation, resulting in two umbilical arteries. Subsequently, each umbilical artery gives rise to a posterior branch, which follows the sciatic nerve (later becoming the inferior gluteal artery), and an anterior branch that traverses the pubic region (developing into the external iliac artery). Later, various branches originate from the internal iliac artery, including the UA [2].

The present study is the first in the literature to showcase an arterial heat map of the UA (Fig. 1), illustrating the topography of this vessel in the pelvic area. The said map demonstrated the most common locations of the origin of the UA and its courses. Our results may aid surgeons in creating a mental map of the uterine vasculature pre- or intraoperatively and can be especially useful when performing high vascular ligation during pelvic and gynecological procedures. Peters et al. [14] introduced an innovative method for precisely identifying the UA during laparoscopic ligation, especially when dealing with distorted pelvic anatomy or variations in the origin of the UA. In a standard hysterectomy, the UA is typically ligated at the level of internal cervical os. However, this traditional approach may become impractical when pelvic pathologies alter the pelvic anatomy. Moreover, the variability in UA origin can further complicate its localization. In response to these challenges, the authors of the study introduced a novel approach to localize the UA at its origin. This can be achieved by approaching the artery either from the pararectal space or by utilizing the medial umbilical ligament, which runs through the paravesical space. Precise knowledge about the anatomy of the UA also relates to protecting the ureter, which passes most commonly under the artery in the pelvis [4]. In the course of treating gynecological pelvic neoplasms necessitating an extensive hysterectomy, there is a potential risk of injuring the ureter when ligating the UA and extending the excision into the parametrium. Injuring this artery may also damage the ureter by decreasing its blood supply [17]. However, the risk of injuring the ureter if the ligation of the UA is performed by its origin, as demonstrated by Peters et al. [14], is reduced immensely. Therefore, effective identification of this artery is of utmost importance. Our study provides useful data for surgeons performing the ligation of the UA by its origin. The surgeon performing this procedure may find the origin of the UA by tracing it from the origin of the anterior trunk of the internal iliac artery. The results of the present study showcase that the distance between these two points is 17.41 mm.

Another technique of hemostasis is the embolization of the UA. This procedure has been used for several gynecological pathologies, including uterine arteriovenous malformations and arteriovenous fistulas, uterine fibroids and leiomyoma, and placenta previa/accreta/percreta [10, 16]. Performing the embolization of the UA to control gynecological hemorrhage effectively is of utmost importance because severe postpartum hemorrhage is an important cause of maternal death and accounts for up to 25% of maternal deaths worldwide [15]. Our study gives valuable data about the topography and morphometric properties of the UA. This data can help physicians perform this endovascular procedure, mainly when choosing the right size of the catheter for the embolization procedure and navigating through the pelvis’s vascular anatomy. Our study demonstrates that the mean diameter of the UA at its origin is 3.37 mm, a relatively higher diameter compared to the recent meta-analysis conducted by Ostrowski et al. (2.73 mm) [13]. Moreover, thorough knowledge regarding the anatomy of the UA prior to its embolization may aid in increasing the efficiency of the procedure, shortening the overall time allocated for finding this vessel [4].

The present study is not without its limitations. Some of the parameters that were initially included in the study protocol were further excluded due to potential bias of the established results. Furthermore, radiological imaging (CTA) can evaluate only hemodynamically efficient arteries. As such, this can be a considerable source of bias when assessing anatomical variations of the UAs and other vascular structures. Additionally, some of the information regarding the patients’ background, for example, history of pregnancies and deliveries, was not gathered. Although not without its limitations, the present study attempts to establish the UA’s detailed morphology and anatomical variations, which meet the requirements of evidence-based anatomy.

## Conclusion

The anatomy of the UA is highly complex, showcasing variable topography, origin patterns, and morphometric properties. In the present study, a novel arterial map of this vessel was made, highlighting the diversity in its origin location and course. In our studied cohort, the UA originated most commonly from the anterior trunk of the internal iliac artery (69.0%), as described in the major anatomical textbooks. Having adequate knowledge about the anatomy of this artery is of immense importance in various gynecological and endovascular procedures, such as hysterectomies and embolizations.

## Data Availability

The data that support the findings of this study are available from the corresponding author, upon reasonable request.
